# Clinical features of low serum alkaline phosphatase levels in children: A retrospective study

**DOI:** 10.1111/ped.70260

**Published:** 2025-11-06

**Authors:** Mami Kurihara, Hanako Tajima, Ryu Ishii, Tae Matsumoto, Makoto Migita

**Affiliations:** ^1^ Department of Pediatrics Nippon Medical School Musashikosugi Hospital Kanagawa Japan; ^2^ Department of Pediatrics Nippon Medical School Hospital Tokyo Japan; ^3^ Department of Pediatrics Nippon Medical School Tamanagayama Hospital Tokyo Japan

**Keywords:** alkaline phosphatase, childhood, hypophosphatasemia, hypophosphatasia, odonto‐hypophosphatasia

## Abstract

**Background:**

Serum alkaline phosphatase (ALP), a biomarker of bone and liver metabolism, is often elevated in children; however, the lower reference limit is rarely considered. Hypophosphatasia (HPP) is characterized by low ALP levels and impaired mineralization of bone and teeth. Although enzyme replacement therapy is available, mild forms are diagnosed late owing to subtle symptoms and ALP levels falling within the adult reference range. In Japan, ALP analysis methods were updated in 2020 and now require value conversion. Pediatricians unfamiliar with these standards may overlook low ALP levels. We investigated whether the disease distribution varies by age and identified key features critical for diagnosis.

**Methods:**

We analyzed serum ALP levels of patients aged <18 years who visited three Nippon Medical School hospitals between January 2020 and December 2022. The inclusion criteria were ALP levels within the adult reference range but below age‐ and sex‐specific pediatric norms. Patient age, sex, and medical history were recorded.

**Results:**

Among 16,125 ALP measurements from 5513 individuals, 239 cases (132 males and 107 females) met the inclusion criteria. In neonates, preterm birth or low birth weight was common, and 55.6% of the infants had infections. School‐age children frequently present with a history of corticosteroid use, while adolescents often exhibit signs of malnutrition or chronic diarrhea. One patient had a prior HPP diagnosis, and two were newly diagnosed.

**Conclusions:**

Our findings underscore the importance of recognizing low ALP levels in pediatric patients and maintaining a high index of suspicion for HPP and other treatable conditions.

## INTRODUCTION

In humans, alkaline phosphatase (ALP) is encoded by four genes: tissue nonspecific (*ALPL*), small intestinal (*ALPI*), placental (*ALPP*), and germline (*ALPG*). Tissue‐nonspecific ALP (TNSALP) is primarily expressed in the liver, bones, and kidneys.^1^, ^2^, ^3^ One of the main roles of TNSALP is to promote bone calcification by hydrolyzing inorganic pyrophosphate (PPi) into inorganic phosphate (Pi), which is a fundamental component of hydroxyapatite.^2^, ^4^ TNSALP deficiency results in impaired bone and tooth mineralization.^2^

ALP levels vary physiologically according to age, sex, and life stage. ALPL is predominantly upregulated during the growth period, and serum ALP levels markedly increase, reaching levels several times higher in children (aged 1–17 years) than in adults (Table 1).^5^, ^6^ Therefore, laboratory reports of ALP levels in children are frequently accompanied by an “H” alert, indicating abnormally high values even when these values fall within the standard range for the child's age and sex. Although pediatricians recognize that the “H” alert for ALP in children does not usually indicate a problem, limited attention has been paid to the lower reference limit. Abnormally low ALP levels in children often fall within the normal range in adults.

**TABLE 1:** Reference values for serum ALP (IFCC method) by age and sex.

	Male	Female
	Range (IU/L)	
0–3 months	175–560	175–560
4–6 months	158–560	140–560
7–12 months	140–525	140–525
1–5 years	140–420	140–420
6–10 years	158–490	158–490
11 years	158–525	140–508
12 years	158–525	105–490
13 years	140–508	70–385
14 years	123–473	70–385
15 years	95–420	53–315
16 years	77–350	46–245
17 years	70–298	42–175
>18 years	38–113	38–113

*Note*: The data were adapted and modified from Reference [5].

Hypophosphatasia (HPP) is a genetic disorder of the *ALPL* gene that results in low serum TNSALP.^1^, ^7^ TNSALP deficiency leads to the accumulation of biological substrates, such as PPi and pyridoxal 5‐phosphate (PLP), resulting in systemic bone and tooth dysplasia. This ultimately leads to impaired bone calcification, ricket‐like changes, and reduced gamma‐aminobutyric acid synthesis.^1^, ^8^, ^9^ The symptoms of HPP include bone fractures, bone pain, arthralgia, and extraskeletal manifestations such as premature tooth loss, ectopic calcification, respiratory failure associated with thoracic hypoplasia, and neurological complications, including vitamin B_6_‐dependent epilepsy.^2^, ^6^, ^8^, ^9^ HPP is classified into six forms according to the age of onset and severity: perinatal severe, perinatal benign, infantile, childhood, adult, and odonto‐hypophosphatasia (teeth‐limited).^1^, ^4^, ^8^ Asfotase alpha, a fusion protein comprising TNSALP, is used for enzyme replacement therapy (ERT) in HPP.^3^ In severe forms, such as perinatal severe and infantile forms, ERT is an essential treatment. In more moderate forms, such as childhood, ERT is used as a relative indication to alleviate bone pain and improve activities of daily living. The early symptoms of mild forms of HPP (childhood, adult, and odonto types) are often nonspecific, and serum ALP levels may only be mildly low, potentially delaying diagnosis. However, early diagnosis and intervention for HPP are recommended in some cases.^4^, ^8^

Low serum ALP levels in children are sometimes overlooked because these values often fall within the normal adult range and do not trigger an “L” alert indicating abnormally low levels.^10^ Early diagnosis of HPP is important because delayed diagnosis can lead to recurrent fractures, bone pain, and a decline in quality of life, although it is treatable.^4^ Moreover, low ALP levels may be linked to various conditions, and recognition in daily practice could help uncover underlying diseases.

Therefore, this retrospective observational study aimed to investigate the clinical conditions and diseases associated with low serum ALP levels in pediatric patients. Specifically, the objectives were to (i) delineate the full spectrum of disorders associated with pediatric hypophosphatasemia, (ii) differentiate between transient and persistent courses of low ALP levels, and (iii) estimate the frequency of HPP among children with low ALP values falling within the adult reference range.

## MATERIALS AND METHODS

### Ethics approval

This study was approved by the Ethics Committee of Nippon Medical School, Tokyo, Japan (approval no. M‐2023‐126). Participants were informed about the study through an opt‐out disclosure process, and consent was obtained accordingly.

### Participants

We retrospectively reviewed the serum ALP data of inpatients and outpatients aged 0–17 years from the Department of Pediatrics at Nippon Medical School Hospital, Nippon Medical School Musashikosugi Hospital, and Nippon Medical School Tama Nagayama Hospital between January 2020 and December 2022. Data were extracted from patients under 18 years of age who had ALP levels within the adult reference range but below the age‐ and sex‐specific pediatric reference values. The reference ranges for children aged <18 years and adults, as defined by the International Federation of Clinical Chemistry and Laboratory Medicine (IFCC), are summarized in Table 1.^6^ Participants were divided into eight groups: neonates (<28 days), infants and toddlers (28 days–5 years), children (6–12 years), and adolescents (13–17 years). Each of the four age groups was analyzed separately by sex (male and female), resulting in eight distinct analysis groups. It is well known that serum ALP levels can increase physiologically during certain life stages, such as puberty and pregnancy. Because the timing of these stages varies considerably among individuals, we chose to use the term “life stage” rather than relying solely on age‐based categorization.

The medical records of the patients were retrospectively reviewed, and data on age, sex, height SD score (SDS), weight SDS, medical history, clinical findings, laboratory data, radiographic findings, medication, and prognosis were collected. In routine clinical practice, we occasionally encounter pediatric cases in which laboratory values fall below age‐ and sex‐specific reference ranges but still fall within the adult upper limit of normal. However, in this study, we focused on conditions that may be overlooked because the test results do not trigger institutional alert thresholds, which are often based on adult reference ranges in many healthcare facilities. Therefore, we adopted this definition to identify such potentially under‐recognized conditions.

### Biochemical analysis

ALP levels were measured using the colorimetric method on IFCC or Japanese Society of Clinical Chemistry (JSCC) analyzers. Traditionally, ALP has been measured in Japan using a unique JSCC method; however, these data are not directly comparable to those obtained using the IFCC method. A nationwide transition from the JSCC method to the IFCC method gradually occurred in 2020. Because this study includes ALP data measured using both methods, ALP levels obtained using the JSCC method were converted to IFCC values by multiplying the JSCC values by 0.35 (IFCC = 0.35 × JSCC).^5^

### Statistical analysis

Patient characteristics, serum ALP levels (IU/L), and medical history were analyzed using descriptive statistics (Microsoft Excel 2021; Microsoft Corporation, Redmond, Washington, USA). If the ALP level was measured multiple times in the same patient, the lowest value was used in the analysis. Additionally, *χ* ^2^ and Cochran–Armitage trend tests were performed to assess the association between categorical variables.

## RESULTS

### Participant characteristics

**FIGURE 1:**
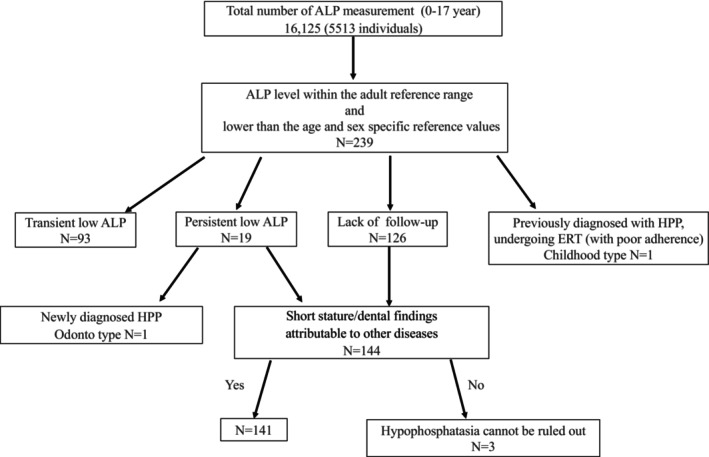
Participants' characters.

**FIGURE 2:**
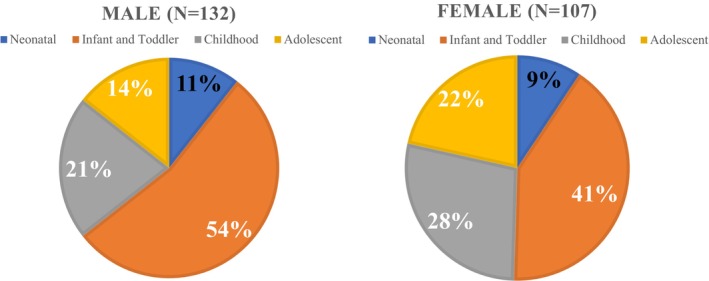
Sex and age distribution of participants. Infants constituted the most frequently represented age group. No significant sex differences were observed across age groups (*p* = 0.18).

**FIGURE 3:**
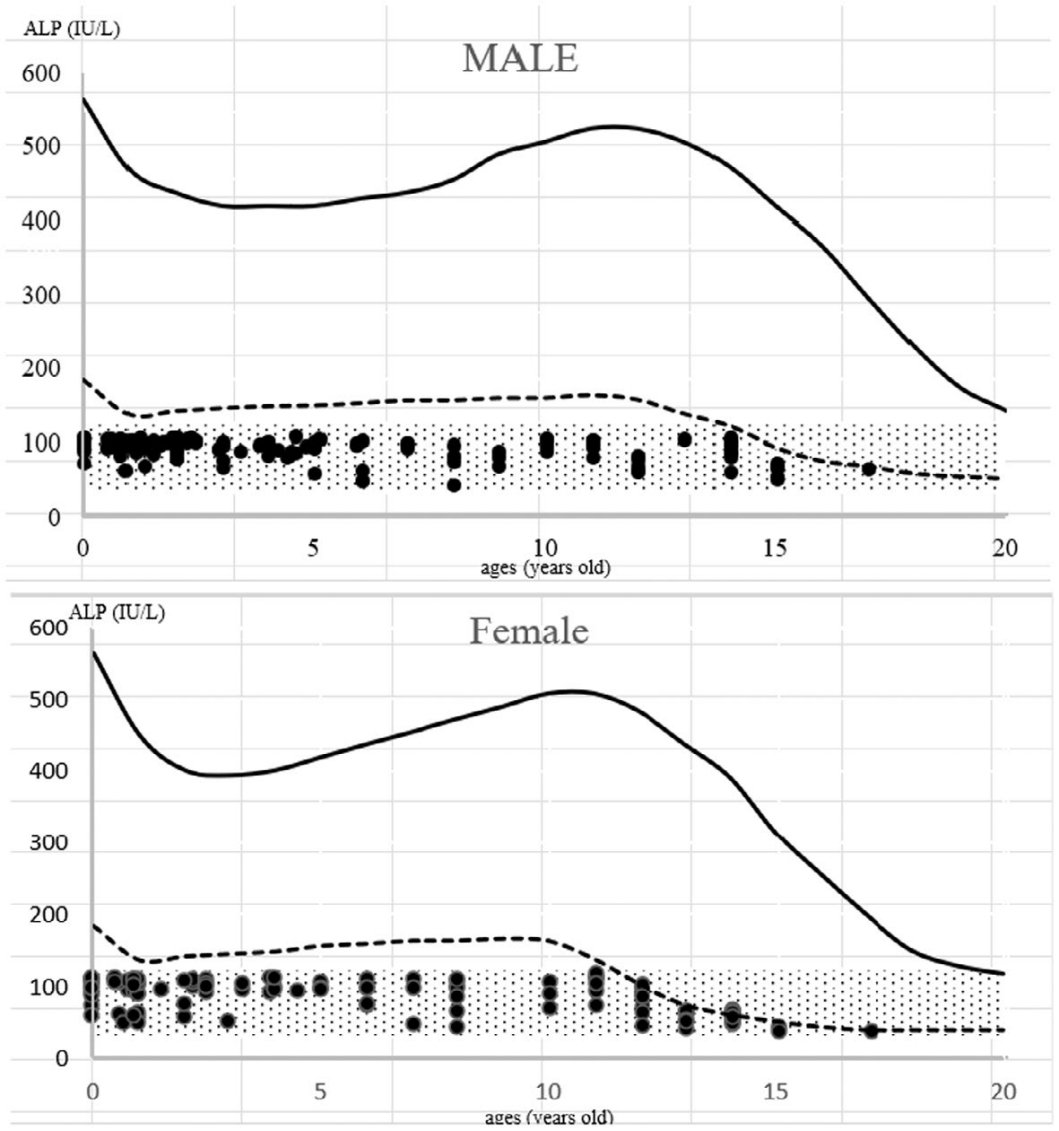
ALP levels according to sex and age. Reference ranges for ALP by age and sex, with ALP values of study participants indicated as black dots. The solid line represents the upper limit of the reference range; the dashed line represents the lower limit. Gray shading indicates adult reference values.

A total of 16,125 ALP measurements were performed in 5513 children aged 0–17 years. Among them, 239 children met the criteria for low ALP levels within the adult reference range and were included in the analysis. A flowchart of the participant inclusion process is shown in Figure 1. The 239 children included 132 males (mean age: 5.1 years) and 107 females (mean age: 6.6 years) (Figure 2). ALP levels according to sex and age are shown in Figure 3. No significant differences were observed between males and females across the different age groups (*p* = 0.18).

**FIGURE 4:**
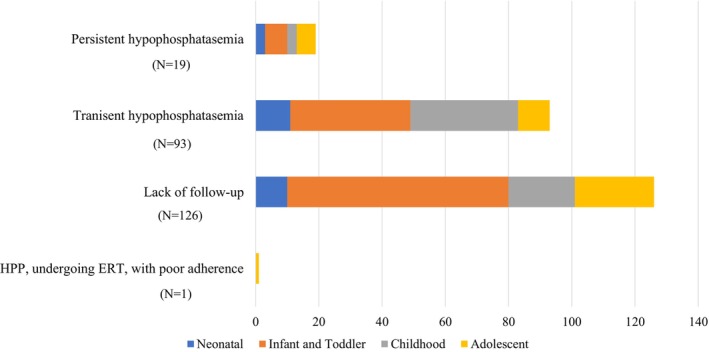
ALP trends among the participants. A large proportion of patients had transient hypophosphatasemia and lack of follow‐up. This was followed by cases of persistent hypophosphatasemia and HPP, undergoing ERT.

To evaluate ALP trends, persistent hypophosphatasemia was defined using a 28‐day interval, consistent with previous reports. However, some cases were followed up at intervals shorter than 28 days.^11^ Transient hypophosphatasemia, defined as low ALP levels that later normalized, was observed in 36.4% of males and 42.1% of females. Persistent hypophosphatasemia was observed in 47.8% of the male patients and 53.8% of the female patients. The remaining cases, in which low ALP levels were detected during the study period but had previously been within the normal range, accounted for 9.1% of males and 10.3% of females (Figure 4). This study included one patient with childhood‐type HPP and one with newly diagnosed odonto‐hypophosphatasia. The patient with childhood‐type HPP had been receiving ERT but continued to show low ALP levels due to poor treatment adherence.

### Disease categories

Diseases were categorized as shown in Table 2. Among patients who exhibited hypophosphatasemia, the proportion receiving corticosteroid therapy was expressed as a percentage. The proportion of affected individuals was calculated to clarify the potential contribution of corticosteroid use to drug‐induced hypophosphatasemia.

**TABLE 2:** Disease trends according to age and sex.

	Neonates (<28 days)		Infants and toddlers (28 days −5 years)		Childhood (6 years‐12 years)		Adolescent (13–18 years)	
	Male	Female	Male	Female	Male	Female	Male	Female
Infectious diseases	0	1	31 (30%)^a^	23 (26%)^a^	3	4	0	2
Hematologic/neoplastic diseases	0	0	4 (0%)^a^	4 (75%)^a^	8 (100%)^a^	6 (83%)^a^	0	4 (50%)^a^
Collagen/rheumatic diseases	0	0	1 (100%)^a^	0	3 (100%)^a^	3 (100%)^a^	2	4 (100%)^a^
Neurological diseases	1	0	3	2	3 (33%)^a^	2	2	2
Gastrointestinal conditions	4	0	1	0	2 (50%)^a^	3	5 (20%)^a^	5
Endocrine/metabolic disorders	1	0	3^b^	0	1	5	2^b^	0
Premature birth and respiratory disorders	7	5	0	0	0	0	0	0
Renal diseases	0	0	5 (100%)^a^	0	2 (100%)^a^	4 (100%)^a^	1 (100%)^a^	0
Vasculitis	0	0	17 (12%)^a^	5 (60%)^a^	1 (100%)^a^	0	0	1 (100%)^a^
Congenital heart diseases	1	1	1	4	0	0	1	0
Postsurgical conditions	0	1	1	0	1	1	2 (50%)^a^	1
Others	0	2	4	7	4 (25%)^a^	3	6	4

^a^ The percentage of patients with suspected corticosteroid‐induced hypophosphatasemia is shown.

^b^ A total of two cases of hypophosphatasia (HPP) were included.

### Disease trends according to age and sex

There were no statistically significant differences between males and females in the following categories: infectious diseases (*p* = 0.81), hematologic/neoplastic diseases (*p* = 0.98), collagen/rheumatic diseases (*p* = 0.88), neurological diseases (*p* = 1.0), gastrointestinal diseases (*p* = 1.0), endocrine/metabolic disorders (*p* = 1.0), premature birth and respiratory disorders in newborns (*p* = 0.94), renal diseases, vasculitis (*p* = 0.31), congenital heart disease (*p* = 0.78), and postsurgical conditions (*p* = 1.0).

Among the neonates included in this study (<28 days of age), the most common conditions were premature birth and respiratory disorders. Other conditions included gastrointestinal issues, such as intestinal malrotation and feeding disorders; cardiac conditions, such as atrioventricular septal defect and premature ventricular contractions; group B *Streptococcus* infection; seizure; hemosiderosis; and following surgery for small bowel obstruction. In neonatal cases, steroid use was not statistically significant (*p* < 0.05).

Among infants and toddlers (28 days–5 years of age), acute illnesses, such as respiratory infections, urinary tract infections, acute focal bacterial nephritis, gastroenteritis, cervical lymphadenitis, and Kawasaki disease, accounted for approximately 70% of cases.

Other diagnoses included malignant tumors acute lymphoblastic leukemia (ALL), acute myeloblastic leukemia (AML), Ewing sarcoma, neuroblastoma, Langerhans cell histiocytosis, and Wilms tumor), juvenile idiopathic arthritis (JIA), immunoglobulin A (IgA) vasculitis, seizures, gastric bleeding, odonto‐hypophosphatasia, nephrotic syndrome, and various congenital heart diseases.

Additional conditions included Down syndrome. Notably, many of these patients received adrenal corticosteroid therapy. The number of cases is presented in Table 2.

In childhood (6–12 years of age), the use of corticosteroids and the incidence of precocious puberty have increased. Diseases requiring adrenal corticosteroid therapy included malignant conditions such as ALL, AML, JIA, systemic lupus erythematosus, nephrotic syndrome, and Kikuchi disease (histiocytic necrotizing lymphadenitis). Other conditions included respiratory infections, gastroenteritis, seizures, irritable bowel syndrome, diabetes mellitus, postoperative recovery after surgery for congenital heart disease, and orthostatic dysregulation. The number of patients with autoimmune diseases and malignant tumors increased with advancing age (*p* < 0.05).

In adolescence (ages 13–17 years), diseases requiring adrenal corticosteroid therapy included malignant conditions such as malignant lymphoma and AML, JIA, systemic lupus erythematosus, IgA nephropathy, and postoperative recovery following surgery for cardiac disease. Other diseases included urinary tract infections, iron‐deficiency anemia, chronic fatigue syndrome, seizures, anorexia nervosa, acute appendicitis, acute pancreatitis, irritable bowel syndrome, precocious puberty, HPP, cardiac disease, postoperative thyroid conditions, postoperative esophageal atresia, orthostatic dysregulation, and school refusal. Significant differences in steroid use were found between patients with and without autoimmune diseases (*p* < 0.05). No statistically significant differences were observed between males and females with respect to autoimmune diseases.

### Participants with HPP

Two patients with HPP (childhood‐type) underwent ERT with asfotase alfa. During ERT, the serum ALP level was typically elevated above the normal range; consequently, one patient did not meet the inclusion criteria. Another patient who underwent ERT with poor adherence exhibited a low serum ALP level during the study period (a 17‐year‐old boy with the lowest ALP level of 52 IU/L). Additionally, two patients whose primary complaint was early exfoliation of deciduous teeth were newly diagnosed with odonto‐hypophosphatasia during the study period. Of these, one patient met the study criteria (4‐year‐old boy, ALP level of 81 IU/L), whereas the other patient (6‐year‐old girl, ALP level of 37 IU/L) did not, as the serum ALP level was below the adult reference range.

## DISCUSSION

This study focused on hypophosphatasemia, defined as ALP values below the normal range for age and sex, but within the normal range for adults. Pediatricians often overlook abnormally low ALP levels within this range. To date, no systematic study has examined hypophosphatasemia in children within the normal adult range. Previous studies have either screened patients with hypophosphatasemia for HPP or enumerated diseases causing hypophosphatasemia^7^, ^10^, ^12^ The frequency of mild adult‐type HPP has been reported to be approximately 1 in 6000 individuals. In this study, one new case was diagnosed among 5513 individuals, which is consistent with previous findings.^4^

In contrast, this study specified the underlying diseases and classified the patients according to their sex and disease type in Japan. The criteria for serum ALP levels in children vary according to factors such as age, sex, life stage, and underlying disease.^5^, ^12^ Several diseases can cause low ALP levels, including metabolic disorders and malnutrition, such as anorexia nervosa, milk allergy, hypomagnesemia, zinc deficiency, celiac disease, and vitamin C or D deficiency.^12^, ^13^ Zinc and magnesium are essential metal cofactors for ALP, which play critical roles in stabilizing the tertiary structure of the enzyme and facilitating its catalytic activity.^14^ Therefore, both zinc and magnesium deficiencies may contribute to the development of hypophosphatasemia. Certain drugs, including corticosteroids, chemotherapeutic agents, and excessive blood transfusions, can also lead to hypophosphatasemia.^12^, ^13^ Glucocorticoids are known to exert direct effects on osteoblasts, suppress bone formation, and consequently lead to decreased serum ALP levels.^15^

In this study, we analyzed the trends in diseases causing hypophosphatasemia according to age group. Among neonates, preterm delivery and respiratory diseases were the most common. However, reports have addressed bone formation and zinc deficiency in preterm infants. Respiratory disorders are common in preterm infants, and hypophosphatasemia may reflect the underlying immaturity of bone formation,^16^ suggesting a potential pathophysiological link between prematurity and hypophosphatasemia. In addition, preterm infants and those born small for gestational age are at an increased risk of zinc deficiency,^17^ which may be associated with secondary hypophosphatasemia. Further studies with larger cohorts are required to clarify this potential relationship.

The following section discusses the findings stratified by age group. Among infants, the incidence of infections was significantly higher in infants than in the other age groups (*p* < 0.05). Corticosteroids were administered to approximately 40% of infants with acute illness, suggesting that hypophosphatasemia may not only result from corticosteroid use but also from inflammation, transient malnutrition associated with appetite loss, or temporary liver dysfunction, all of which reduce ALP production. Intestinal ALP (IAP) has been implicated in intestinal immunity related to ALP.^18^ IAP activity has been shown to correlate with IgA levels in the intestinal tract of mice, and is positively correlated with IgA, IgG, and IgM levels in human stool.^19^ These findings underscore the important role of IAP in acquired immunity.^20^ Patients with chromosomal abnormalities are prone to insufficient oral intake and are at an increased risk of malnutrition.

In childhood, all the children with autoimmune or malignant diseases in the present study received corticosteroids. Although the mechanism underlying hypophosphatasemia induced by chemotherapeutic agents other than corticosteroids has not yet been elucidated, these agents are frequently associated with bone marrow suppression and hepatic dysfunction, which may indirectly influence serum ALP activity. In patients with precocious puberty, ALP levels are elevated in the early stages but may decline to relatively low levels once secondary sexual characteristics are fully developed at an earlier age.^21^ Postoperatively, endotoxemia can also cause low ALP levels.^22^ Endotoxemia is particularly common both before and after infant cardiothoracic surgery with cardiopulmonary bypass^23^, ^24^ The inverse relationship between ALP activity and endotoxemia, as well as the ex vivo reduction in endotoxin activity with exogenous ALP supplementation, suggests that ALP plays a role in mitigating endotoxemia in children undergoing cardiopulmonary bypass.^22^

Adolescents often present with malnutrition‐related issues, such as eating disorders, iron‐deficiency anemia, orthostatic dysregulation, and school non‐attendance.^15^ Although patients with iron‐deficiency anemia can develop low ALP levels, the underlying mechanism remains unclear. Anemia is often accompanied by malnutrition, including zinc deficiency or hypomagnesemia, which are essential cofactors for activating ALP. Findings from a study on children with protein‐energy malnutrition indicated that zinc deficiency secondary to undernutrition may impair the activity of antioxidant and metabolic enzymes, thereby contributing to reduced ALP activity.^25^ Hypophosphatasemia can also occur after thyroid surgery or in patients with hypothyroidism. Thyroid hormones stimulate osteoblast activation. Both bone formation and resorption are reduced in hypothyroidism, leading to a decreased production of bone‐derived ALP.^23^, ^24^, ^26^

An age‐related increase in the number of patients with malignancies and autoimmune diseases has been observed, among whom corticosteroid use is common. In addition to corticosteroid therapy, a higher prevalence of malignancies and chronic diseases may contribute to low ALP levels through mechanisms such as malnutrition, bone metabolism disorders, and chronic inflammation. Therefore, the underlying factors associated with hypophosphatasemia may vary by age, and both age and comorbid conditions should be considered when interpreting ALP levels.

Hypophosphatasemia can be classified into categories based on temporal changes in serum ALP levels.^3^ Persistently low ALP levels are associated with conditions such as HPP, osteogenesis imperfecta, and cleidocranial dysplasia. Transiently low ALP levels are often observed in conditions such as hypothyroidism, Cushing's disease, bisphosphonate therapy, renal osteodystrophy, milk‐alkali syndrome, vitamin D intoxication, Wilson's disease, hypomagnesemia, zinc deficiency, vitamin C deficiency, celiac disease, and pernicious anemia. Finally, precipitously low ALP levels are observed in critical situations, such as major trauma, major surgery, cancer, chemotherapy, conditions necessitating massive blood transfusions, starvation, sepsis, multiorgan or hepatic failure, and improperly collected specimens (e.g., those contaminated with ethylenediaminetetraacetic acid, citrate, or oxalate).^13^ Several mechanisms have been proposed to explain the transient hypophosphatasemia observed in children with acute illnesses.

First, nutritional deficiencies, particularly reduced intake during illness, can lead to temporary insufficiency of essential cofactors, such as zinc and magnesium, which are required for ALP synthesis. Second, suppression of osteoblastic activity, caused by systemic stress, corticosteroid use, or proinflammatory cytokines such as IL‐6 and TNF‐α, may result in reduced ALP production. These cytokines have been shown to inhibit ALP gene expression in osteoblasts and hepatocytes under certain conditions.^27^ These factors may act synergistically to contribute to the transient decrease in serum ALP levels. Longitudinal monitoring of ALP trends, along with simultaneous evaluation of hypothyroidism and zinc deficiency, is recommended to help identify the underlying cause of hypophosphatasemia.^28^ Among the 239 patients included in this study, 144 had persistent low ALP or lack of follow‐up. Five patients exhibited short stature (height <−2 SD); of these, three could not be explained by endocrinological, metabolic, or congenital anomaly syndromes. Although none of these patients exhibited classic signs of HPP (fractures or early loss of deciduous teeth), hypophosphatasia (atypical or childhood) could not be entirely ruled out without further investigation.

In such cases, it is recommended to obtain a detailed history of the early exfoliation of deciduous teeth, reassess dental findings, and recheck serum ALP levels during future visits.

In Japan, the method for measuring serum ALP levels changed from the JSCC method to the IFCC method in 2020, which also resulted in a change in the standards for serum ALP levels. During the transition period, some pediatricians in Japan may not have been fully accustomed to the new ALP standards, potentially leading to the oversight of cases with low serum ALP levels. Our facility implemented an alert system for abnormal ALP levels by 2022. This system notifies clinicians of abnormal ALP levels based on age and sex and has been particularly helpful for pediatricians in identifying abnormally low ALP levels.

This study had several limitations, including the small number of participants, the lack of follow‐up data on serum ALP levels in 126 of 239 cases, and the absence of isozyme analysis, confounding factors associated with low ALP levels, such as serum zinc, and markers of malnutrition, in most cases with low ALP levels. Additionally, this study may be subject to selection bias due to its setting in a tertiary care center, and the lack of growth velocity data may have led to underestimation of the effects on skeletal development. Furthermore, this study did not confirm the effect of hypophosphatasemia in these patients. Careful follow‐up of patients with hypophosphatasemia is essential because many diseases or conditions can be associated with low serum ALP levels. This study did not distinguish between transient and persistent hypophosphatasemia, and it was not discussed in this study. The potential for confounding by drugs, nutritional status, and disease severity also needs to be explored. Future studies should be conducted to expand the sample size, measure serum calcium and phosphate levels, and gather additional prospective data. Moreover, ALP isoenzyme levels were not measured in all the patients. Among the cases in which isoenzyme analysis was performed, no marked decrease in intestinal ALP was observed. Future studies should aim to expand the sample size, assess serum calcium and phosphate levels, and collect prospective data to further investigate the underlying mechanisms and clarify the causes of hypophosphatasemia.

In conclusion, the causes of hypophosphatasemia in children vary with age and include physiological changes, malnutrition, inflammation, endocrine disorders, and drug‐induced factors. This study underscores the importance of recognizing low ALP levels that fall within the adult reference range but are below age‐ and sex‐specific pediatric standards. Such values are often overlooked; however, they may serve as early indicators of underlying conditions, including metabolic bone diseases. Differentiating the underlying causes of low ALP levels and conducting further assessments, such as evaluating growth impairment, reassessing the exfoliation of deciduous teeth, and considering ALP isoenzyme analysis when appropriate, may facilitate earlier and more accurate diagnosis of relevant conditions, including hypophosphatasia.

## AUTHOR CONTRIBUTIONS

H.T., M.M., and M.K. designed the study; M.K., H.T., R.I., M.T., and M.M. performed the experiments; M.K., H.T., R.I., and T.M. collected and analyzed the data; M. K. and H. T. wrote the manuscript; H. T., T. M., and M. M. provided technical support and conceptual advice.

All authors confirmed and approved the final manuscript.

## FUNDING INFORMATION

This research was funded by the Support Program for Research Activities of Female Researchers, Japan Science and Technology Agency, and Ministry of Education, Culture, Sports, Science, and Technology.

## CONFLICT OF INTEREST STATEMENT

The authors declare no conflict of interest.

## Data Availability

The data that support the findings of this study are available on request from the corresponding author. The data are not publicly available due to privacy or ethical restrictions.
